# The occurrence of long COVID: a rapid review

**DOI:** 10.11604/pamj.2021.38.65.27366

**Published:** 2021-01-20

**Authors:** Chinwe Juliana Iwu, Chidozie Declan Iwu, Charles Shey Wiysonge

**Affiliations:** 1 Department of Nursing and Midwifery, Faculty of Medicine and Health Sciences, Stellenbosch University, Cape Town, South Africa,; 2 School of Health Systems and Public Health, Faculty of Health Sciences, University of Pretoria, Pretoria 0001, South Africa,; 3 Cochrane South Africa, South African Medical Research Council, Cape Town, South Africa,; 4 Department of Global Health, Stellenbosch University, Cape Town, South Africa,; 5 School of Public Health and Family Medicine, University of Cape Town, Cape Town, South Africa

**Keywords:** Long COVID, long haulers, long term effects of COVID, post-COVID syndrome, SARS-CoV-2, systematic review

## Abstract

The long-term effects of the severe acute respiratory syndrome (SARS) coronavirus 2 (SARS-CoV-2) are not well understood. This rapid review was aimed at synthesizing evidence on the long-term effects of the SARS-CoV-2 infection among survivors. We considered both randomised controlled trials and non-randomised studies eligible for inclusion in this review. The following databases were searched: PubMed, Scopus, Cochrane library, Google Scholar, and the World Health Organization (WHO) COVID-19 database. The reference lists of all the included studies were also searched. Two authors independently screened the search outputs and reviewed full texts of potentially eligible articles. Data extraction was done by one author and checked by a second author. A meta-analysis was not conducted due to heterogeneity among the included studies. Results are presented narratively. Eleven studies met our inclusion criteria. All these studies were conducted in high-income countries. Study findings demonstrate that COVID-19 survivors can experience persistent symptoms after recovering from their initial illness, especially among previously hospitalized persons. The majority of symptoms reported were fatigue, shortness of breath, cough, and sleep disorders. Mental conditions, such as depression and anxiety disorders, were also reported. In conclusion, this study showed that COVID-19 survivors can experience persistent symptoms after recovering from their initial illness. Therefore, there is a need for a long-term follow-up of COVID-19 patients and rehabilitation services for survivors. More research is needed in this area, especially in Africa.

## Introduction

The present coronavirus disease (COVID-19) pandemic has continued to pose a severe threat to the global community. Caused by the novel severe acute respiratory syndrome (SARS) coronavirus 2 (SARS-CoV-2), the disease has proven to be an unprecedented health challenge affecting all forms of human existence [1]. The clinical spectrum of SARS-CoV-2 infection has been shown to range from asymptomatic infection to critical illness [2]. The median incubation period among symptomatic patients is approximately 4 to 5 days. Close to 98% of these patients develop symptoms within 11.5 days after infection [3]. The common symptoms may include fever, cough, sore throat, malaise, and myalgias. Some patients present with gastrointestinal symptoms, including anorexia, nausea, and diarrhea. Anosmia and ageusia have also been reported, almost 70% of patients. Shortness of breath is also common, which suggests worsening disease [3]. However, data have recently emerged that some patients continue to experience symptoms related after the acute phase of infection [4]. This phenomenon is regarded as “long COVID”; it describes the illnesses in people who have recovered from COVID-19 or the unusual symptoms of COVID-19 that have persisted longer than expected [5].

**Review objective:** the purpose of this paper is to systematically review the available literature on long COVID. COVID-19 is a new disease and it will be important to understand its long-term effects on survivors.

## Methods

This review was reported according to the preferred reporting items for systematic review and meta-analyses (PRISMA) guideline [6].

**Eligibility criteria:** the following study designs were eligible for inclusion in this review, randomized trials (with randomization at either individual or cluster levels), case-controlled studies, case reports, case series prospective cohorts, controlled before-after studies, interrupted time series studies, and repeated cross-sectional studies, with no restrictions on language and publication status. The target population was both adults and children. Our primary outcomes of interest were symptoms reported by authors of articles, including persistent or new symptoms.

**Search methods for identification of studies:** we developed a comprehensive and search strategy for peer-reviewed studies and grey literature. We systematically searched the following databases from inception to November 23^rd^, 2020: the Cochrane Library, PubMed, and Scopus. We also explored the World Health Organization (WHO) COVID-19 database and Google scholar. We screened the reference lists of all the included studies and related reviews for potentially eligible primary studies. The keywords used include “post-COVID-19 syndrome”, “long haulers,” “long hauling,” and “chronic COVID syndrome.” Only primary studies were considered eligible for inclusion. Table 1 contains search strategy conducted for both PubMed and Scopus. Two authors (CDI and CJI) independently screened through titles and abstracts of the retrieved records to identify potentially eligible studies. The full texts of these potentially eligible studies were also assessed using the pre-specified eligibility criteria. The two authors compared lists of included studies and resolved discrepancies by discussion and consensus. The reference lists of included studies, including literature reviews not included in this review, were checked for any eligible studies.

**Table 1:** search strategy for search conducted on November 23^rd^, 2020

Search string	Results
PubMed	
“post COVID-19 syndrome”[Title/Abstract] OR “long haulers”[Title/Abstract] OR “long hauling”[Title/Abstract] OR “long COVID”[Title/Abstract] OR “chronic COVID syndrome”[Title/Abstract]	12
Scopus	
TITLE-ABS-KEY (“post COVID-19 syndrome” OR “long haulers” OR “long hauling” OR “long COVID” OR “chronic COVID syndrome”)	53

**Data collection and analysis:** a data collection form was designed and used by one review author (CJI) and independently checked by another review author (CDI) for accuracy. The following information was extracted from each included study; study setting (country), type of study, study participants, outcome measures, and study findings. We didn't contact authors for missing data, as all data required to complete the review were available within published articles. Since this is a rapid review, we didn't assess the risk of bias for the included studies. Data were synthesized narratively.

**Patient and public involvement:** patients or the public were not involved in the design, or conduct, or reporting, or dissemination plans of our research.

## Current status of knowledge

**Figure 1:**
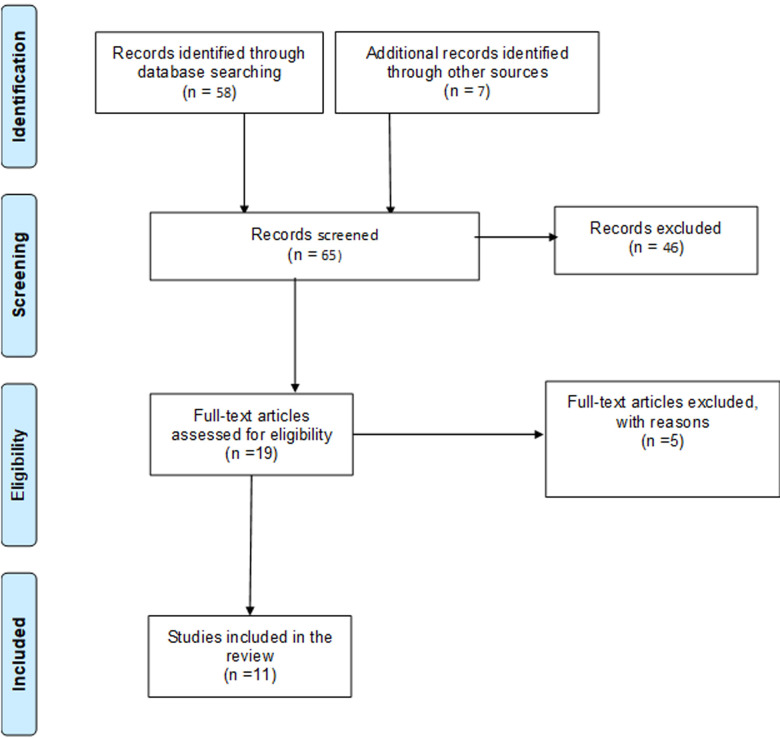
PRISMA flow diagram of study selection for a rapid review on the occurrence of long COVID

**Results of the search:** the literature search from all sources yielded a total of 65 articles. The titles and abstracts of these articles were screened for potentially eligible studies. Forty six articles were excluded after the title and abstract screening. The full texts of 19 potentially eligible articles were therefore assessed for eligibility. Eleven studies were finally included in this rapid review [7-14], while five studies were excluded after full-text screening [15-19], as they were all short communications and commentaries. Figure 1 describes the entire process of study selection. These studies were mostly conducted in high-income settings, namely USA [7,13,20,21] United Kingdom [10,12,22] and Europe (Sweden, France, Italy, and Austria) [8,9,11,14]. No study was conducted in low- and middle-income countries, including Africa. Majority (six) of the studies were conducted using surveys [7-12] while two studies were case reports [13,22]. Of the six surveys, two were conducted telephonically [11,12]. Table 2, Table 2 (suite), Table 2 (suite 1) summarise the characteristics of the 11 included studies.

**Table 2:** characteristics of included studies for the review on long COVID

Author	Country	Population	Study design	Outcome measures	Study findings (reported symptoms)
Mandal 2020	UK	384 patients mean age of 59.9 years and predominantly male. 34% of these patients had no reported comorbidity; 43% were from a black, Asian or minority ethnic background; 8% were obese.	Telephonic survey 4 to 6 weeks after discharge	Persistent symptoms	53% reported persistent breathlessness, 34% persistent cough and 69% persistent fatigue. Fifteen per cent were depressed. Poor sleep quality was also reported Blood tests showed that 7.3% of 247 patients had persisting Lymphopaenia; 30.1% of 229 had elevated d- dimer and 9.5% of 190 patients had elevated C reactive protein (CRP).
Ludvigsson 2020	Sweden	Five Swedish children; four girls one boy. Average age was 12 years. Children had experienced symptoms for between six and eight months Only one child had comorbidities before developing COVID-19; a 12-year-old female with asthma, allergies and mild autism spectrum disorder	Case report	Persistent symptoms	All had fatigue, dyspnoea, heart palpitations or chest pain. Four had headaches, difficulties concentrating, muscle weakness, dizziness and sore throats. Parents also reported that three of the children experienced abdominal pain, memory loss, depression and skin rashes and muscle pain Less common symptoms, experienced by two children, were remitting fever, sleep disorders, joint pain, diarrhoea and vomiting
Tenforde 2020	USA	Symptomatic adults (18-34 years) who had a positive out-patient test result for SARS-CoV-2 infection	Summary of reports of studies on post-COVID-19 syndrome survey	Return to usual state of health	35% had not returned to their usual state of health when interviewed 2-3 weeks after testing. Among persons aged 18-34 years with no chronic medical conditions, one in five had not returned to their usual state of health.
Carvalho-Schneider 2020	France	150 patients with non-critical COVID-19 on days 7, 30 and 60 after confirmation	Descriptive clinical follow-up study	Persistent symptoms	Two-thirds of adults experienced persistent symptoms up to 2 months after symptom onset, primarily anosmia and ageusia, dyspnoea or fatigue.
Garrigues 2020	France	Previously hospitalized patients; 120 patients were included in the study (96 who had not been in the ICU and 24 who had been in the ICU). The mean age was 63.2 (standard deviation, 15.7 days), and 62.5% were male	A follow-up phone survey	Post discharge clinical symptoms among hospitalized patients	After a mean of 110.9 days, the most frequently reported persistent symptoms were fatigue (55%), dyspnoea (42%), loss of memory (34%), concentration and sleep disorders (28% and 30.8%, respectively).
Carfi 2020	Italy	143 patients. The mean age was 56.5 years; 37% were women and 10% were active smokers	Survey	Persistent symptoms	Most common symptoms were fatigue (53%), dyspnea (43%), arthralgias (27%) and chest pain (22%).
Halpin 2020	UK	100 patients with COVID-19 discharged from the hospital at least 4 weeks prior to study enrollment; 32 patients had been in the ICU	Telephonic survey	Patients' symptoms post-discharge and the impact on their daily life	New fatigue was the most reported symptom. 72% of participants in the ICU group and 60.3% non-ICU group reported fatigue
European Respiratory Society 2020	Austria	86 patients followed up, 6 weeks, and 12 weeks after discharge from hospital. Average age was 61 years. Nearly half of them were current or former smokers and 65% of hospitalised COVID-19 patients were overweight or obese. Eighteen (21%) had been in an intensive care unit (ICU), 16 (19%) had had invasive mechanical ventilation, and the average length of stay in hospital was 13 days.	Prospective cohort	Clinical examinations, laboratory tests, analysis of the amounts of oxygen and carbon dioxide in arterial blood, lung function tests, computed tomography (CT) scans. Tests of lung function included FEV1 (the amount of air that can be expelled forcibly in one second), FVC (the total volume of air expelled forcibly), and DLCO (a test to measure how well oxygen passes from the lungs into the blood).	A total of 56 patients (65%) showed persistent symptoms at the time of their six-week visit; dyspnoea was the most common symptom (40 patients, 47%), followed by coughing (13 patients, 15%). CT scans still showed lung damage in 88% of patients during the six weeks visit. By the next visit 12 weeks after discharge, the symptoms cough and breathlessness had improved; 13 patients (15%) were still coughing and dyspnoea was present in 31 patients (39%). The lung damage was reduced from 88% to 56%. Biological indicators of heart damage, blood clots and inflammation were all significantly elevated.
Tacquet 2020	USA	Patients older than 10 years	large federated electronic health record network in the USA known as the TriNetX Analytics Network that captures anonymised data from electronic health records in 54 health-care organisations in the USA, totalling 69.8 million patients.	incidence of a first psychiatric diagnosis, over a period from 14 days to 90 days after a diagnosis of COVID-19, as well as insomnia and dementia	In patients with no previous psychiatric history, a diagnosis of COVID-19 was associated with increased incidence of a first psychiatric diagnosis in the following 14 to 90 days compared with six other health events. The most frequent psychiatric diagnosis after COVID-19 diagnosis was anxiety disorder (HRs 1.59-2.62, all p<0.0001), with a probability of outcome within 90 days of 4.7% (95% CI 4.2-5.3). with adjustment disorder, generalised anxiety disorder, and, to a lesser extent, post- traumatic stress disorder being the most frequent. Other notable disorders were mood disorder, insomnia, and dementia. Dementia was noted in patients 65 years and older.
Weerahandi 2020	USA	A total of 161 patients 18 years and older who were hospitalized with laboratory-confirmed COVID-19 disease. Median age was 62 years. 57 (37%) were female. These patients required at least 6 liters of oxygen during admission, had intact baseline cognitive and functional status and were discharged alive. Participants	Prospective single health system observational cohort study one month after discharge for severe COVID-19	Outcomes were elicited through validated survey instruments: the PROMIS® Dyspnea Characteristics and PROMIS® Global Health-10 This instrument measured the overall health, physical health and mental health of patients	152 (38.3%) completed the survey: 113/152 (74%) participants reported shortness of breath within the prior week (median score 3 out of 10 [IQR 0-5]), vs. 47/152 (31%) pre-COVID-19 infection (0, IQR 0-1), p<0.001. Participants also rated their physical health and mental health as worse in their post-COVID state (43.8, standard deviation 9.3; mental health 47.3, SD 9.3) compared to their pre-COVID state, (54.3, SD 9.3; 54.3, SD 7.8, respectively), both p<0.001. A total of 52/148 (35.1%) patients without pre-COVID oxygen requirements needed home oxygen after hospital discharge; 20/148 (13.5%) reported still using oxygen at time of survey.
Novak 2020	USA	A 64-year-old woman presented with a cough and dyspnea.	Case report	Vascular changes	Chronic fatigue, orthostatic dizziness and brain fog consistent with orthostatic hypoperfusion syndrome (OCHOS). The patient has a history of these conditions. However, she was stable prior being infected with the virus and developed these symptoms two weeks after the symptoms of COVID-19 (fever and respiratory symptoms) had disappeared. The patient recovered after immunotherapy. Implying that COVID-19 can trigger autoimmunity and may have exacerbated the condition.

**Study characteristics:** in 10 of the 11 included studies [7-14,20,21] (Table 2, Table 2 (suite), Table 2(suite 1)), the participants were adults between 18 and above 60 years [6-12], only one study comprised children whose average age was 12 years [22] (Table 2). These patients were all symptomatic during the active infection, ranging from mild to severe and hospitalized cases. The hospitalized patients also included those who were admitted to the intensive care units (ICUs). Outcome measures reported in the studies were mostly persistent symptoms [8-12,22] (Table 2, Table 2 (suite), Table 2 (suite 1)). Other outcomes measured were impact on daily life [10] (Table 2 (suite)); vascular changes [13] (Table 2 (suite 1)); return to usual state of health (Table 2) [7]; incidence of a first psychiatric diagnosis (Table 2 (suite)) [20]; overall health, physical health and mental health of patients (Table 2 (suite 1)) [21]. The common persistent symptoms described in these studies include fatigue in six studies (Table 2, Table 2 (suite)) [8-12,22], difficulty in breathing in five studies (Table 2, Table 2 (suite)) [8,11,12,14,22], cough in three studies (Table 2,Table 2 (suite 1)) [12-14]; chest pain in two studies (Table 2) [9,22], sleep disorders in four studies (Table 2, Table 2 (suite)) [11,12,20,22] loss of memory in two studies (Table 2) [11,22]; muscle pain and weakness in two studies (Table 2) [9,22]. Other symptoms reported include: heart palpitations, headaches, difficulties concentrating, muscle weakness, dizziness, sore throats, loss of sense of smell (anosmia), loss of sense of taste (ageusia), skin rashes, hair loss, diarrhoea and vomiting. Lung damage and heart dysfunction were reported in one of the studies, which was conducted in Austria (Table 2 (suite)) [14]. In this study, 86 patients were followed up, 6 weeks, and 12 weeks after discharge from hospital. At 6 weeks, computed tomography (CT) scans showed lung damage in 88% of patients. However, by the 12^th^ week after discharge, the lung damage was reduced from 88% to 56% and the symptoms cough and breathlessness had improved.

There was significant elevation of biological indicators of heart damage, blood clots and inflammation six weeks after discharge from the hospital in the study conducted in Austria (Table 2 (suite)) [14]. Similarly, another study conducted in the UK, showed that 7.3% of 247 patients had persisting lymphopaenia; 30.1% of 229 had elevated D-dimer and 9.5% of 190 patients had elevated C reactive protein (CRP), in addition to other persisting symptoms such as difficulty in breathing, cough and fatigue depression and sleep disorders (Table 2) [12]. Lymphopaenia and elevated d-dimer and C reactive protein (CRP) are indicative of indicative of reduced immunity, clotting disorder and ongoing inflammation respectively. Our study also showed that COVID-19 could lead to psychiatric illnesses. For example, the study which looked at the psychiatric sequalae of COVID-19 during the first 14 to 90 days after a diagnosis, demonstrated that in patients with no previous psychiatric history, a diagnosis of COVID-19 was associated with an increased incidence of a first psychiatric diagnosis (Table 2 (suite)) [20]. The most frequent psychiatric diagnosis was anxiety disorder, including adjustment disorder, generalised anxiety disorder, and, to a lesser extent, post-traumatic stress disorder. Other notable disorders were mood disorder, insomnia, and dementia. The latter was noted in patients 65 years and older. The probability of being newly diagnosed with a psychotic disorder was low. Another study suggested that long COVID may present as an autoimmune orthostatic hypoperfusion syndrome (OCHOS). A case report from the USA showed that a 64-year-old woman presented chronic fatigue, orthostatic dizziness and brain fog consistent with OCHOS. Although the patient has a history of this condition, she was stable before being infected with the virus. She developed these symptoms two weeks after the symptoms of COVID-19 (fever and respiratory symptoms) had disappeared. Authors thought that COVID-19 might have triggered this condition (Table 2 (suite)) [13].

**Summary of main findings:** this study was aimed at determining the occurrence of long effects of COVID-19. We found 11 studies eligible for inclusion in this review. Most studies reported on symptoms persisting even after recovery or discharge from the hospital. The majority of symptoms reported were fatigue, shortness of breath, cough, and sleep disorders. Symptoms include loss of memory, muscle pain and weakness, heart palpitations, headaches, difficulties concentrating, dizziness, sore throat, loss of sense of smell, loss of sense of taste, skin rashes, hair loss, diarrhoea, and vomiting were also reported in the studies included. Psychiatric illnesses, including anxiety disorders, were also reported. This implies that COVID-19 could have long term effects on both the physical and mental state of survivors. Furthermore, our study also showed that COVID-19 could lead to persistent low immunity, clotting disorders, and inflammation. Both heart and lung damages are also possibilities, as demonstrated from the findings of this study. A study has shown that the SARS-CoV-2 virus induces vascular inflammation, which may be responsible for persisting symptoms seen among COVID-19 survivors [2].

Long term effects of the SARS virus have been reported, with similar symptoms reported for the SARS-CoV-2 virus. Evidence from the previous SARS epidemic also suggests that these symptoms can last for many years. For example, symptoms such as fatigue, muscle pain, sleep disorders, and depression were noted in a case-controlled study of people previously infected by the SARS virus in Canada [23]. Another study that tracked people with SARS for four years in China found 40% had chronic fatigue [24]. A study in China showed reduced lung function, exercise capacity, and respiratory muscle strength in SARS survivors, 24 months after assessment [25]. Lung damage noted in one of the included studies [13] was also reported among patients infected by the SARS virus. This study showed that 4.6% of 71 SARS survivors still had visible lesions and 38% had reduced diffusion capacity 15 years after being discharged from the hospital [26]. It has been suggested that some of the damage to the lungs is likely to be a side effect of intensive treatments such as intubation, coupled with the lingering problems that could be caused by the virus itself [26].

**Implications for policy and research:** this study's findings show the need for implementing structured follow-up care for COVID-19 survivors, especially those who were hospitalized and those with underlying co-morbidities. The follow-up might help preempt and treat symptoms that could severely impact the health of survivors [14]. As the number of persons suffering from the long-term effects of COVID-19 increases, there is also a need to develop clinical guidelines on the care of survivors of COVID-19 [26]. It is also vital for more studies to be conducted in this area, especially in Africa. Well-designed studies such as prospective cohort studies of patients with COVID-19 with adequate control groups will particularly be useful. Studies that could demonstrate the underlying mechanisms behind these long-term effects could improve the clinical management of these symptoms.

**Study limitations:** the quality of the included studies was not described in this review since the risk of bias assessment for included studies was not done. In addition, the topic of long COVID is new, and there is no consensus yet on a name used to describe it. Hence some articles may have been missed during selection due to the differences in the terms and how they are indexed in the databases.

## Conclusion

This rapid review shows that COVID-19 survivors can experience persistent symptoms after recovering from their initial illness. Major symptoms reported were: fatigue, shortness of breath, cough, and sleep disorders. Other symptoms reported include loss of memory, muscle pain, weakness, heart palpitations, headaches, difficulties concentrating, dizziness, sore throat, loss of smell, loss of taste, skin rashes, and hair loss, diarrhoea, and vomiting. Psychiatric illnesses, including anxiety disorders, were also reported. Furthermore, our study also showed that COVID-19 could lead to persistent low immunity, clotting disorders, and inflammation. COVID-19 survivors could experience mental conditions, such as depression and anxiety disorders. Damage to the lungs and heart could occur, but the extent and duration of this damage need to be further substantiated. There is, therefore, a need for a long-term follow-up of COVID-19 patients coupled with rehabilitation services to those with persistent or new symptoms. There is also a need for more research in this area, especially in other regions, particularly the low-and-middle-income countries, including Africa. Such research will be important as it will help unravel possible underlying mechanisms causing long-term effects of COVID-19, reasons why symptoms persist or recur, and prognosis.

### What is known about this topic

- The clinical spectrum of SARS-CoV-2 infection has been shown to range from asymptomatic infection to critical illness requiring hospitalization;
- The long-term effects of SARS-CoV-2 infection are not well understood.

### What this study adds

- There is scarcity of published studies on long COVID in Africa;
- Study findings demonstrate that COVID-19 survivors can experience persistent COVID-19 symptoms after recovering from their initial illness. Common symptoms of long COVID include fatigue, shortness of breath, cough, and sleep disorders. Other symptoms that could occur include, loss of memory, muscle pain, weakness, heart palpitations, headaches, difficulties concentrating, dizziness, sore throat, loss of smell, loss of taste, skin rashes, and hair loss, diarrhoea, and vomiting;
- COVID-19 survivors could also experience mental conditions, such as depression and anxiety disorders. Damage to the organs, such as the lungs and heart could also occur.
